# Neuroprotection during Thrombectomy for Acute Ischemic Stroke: A Review of Future Therapies

**DOI:** 10.3390/ijms25020891

**Published:** 2024-01-10

**Authors:** Vikalpa Dammavalam, Sandra Lin, Sayedatun Nessa, Neil Daksla, Kamil Stefanowski, Ana Costa, Sergio Bergese

**Affiliations:** 1 Department of Neurology, Stony Brook University Hospital, Stony Brook, NY 11794, USA; vikalpa.dammavalam@stonybrookmedicine.edu (V.D.); kamil.stefanowski@stonybrookmedicine.edu (K.S.); 2 Department of Anesthesiology, Stony Brook University Hospital, Stony Brook, NY 11794, USA; sandra.lin@stonybrookmedicine.edu (S.L.); neilchristopher.daksla@stonybrookmedicine.edu (N.D.); ana.costa@stonybrookmedicine.edu (A.C.)

**Keywords:** acute ischemic stroke, thrombectomy, neuroprotection

## Abstract

Stroke is a major cause of death and disability worldwide. Endovascular thrombectomy has been impactful in decreasing mortality. However, many clinical results continue to show suboptimal functional outcomes despite high recanalization rates. This gap in recanalization and symptomatic improvement suggests a need for adjunctive therapies in post-thrombectomy care. With greater insight into ischemia-reperfusion injury, recent preclinical testing of neuroprotective agents has shifted towards preventing oxidative stress through upregulation of antioxidants and downstream effectors, with positive results. Advances in multiple neuroprotective therapies, including uric acid, activated protein C, nerinetide, otaplimastat, imatinib, verapamil, butylphthalide, edaravone, nelonemdaz, ApTOLL, regional hypothermia, remote ischemic conditioning, normobaric oxygen, and especially nuclear factor erythroid 2-related factor 2, have promising evidence for improving stroke care. Sedation and blood pressure management in endovascular thrombectomy also play crucial roles in improved stroke outcomes. A hand-in-hand approach with both endovascular therapy and neuroprotection may be the key to targeting disability due to stroke.

## 1. Introduction

The global incidence of ischemic strokes is estimated at 7.6 million per year [1]. In addition to increased focus on risk factor modification, recent groundbreaking advances in acute ischemic stroke (AIS) treatment were impactful in decreasing mortality rates [2,3]. Consequently, the prevalence of individuals afflicted with disability due to stroke has increased, making stroke the leading cause of long-term disability worldwide [4]. Growing efforts in post-stroke care with neuroprotective agents may have the potential to mitigate the disability associated with ischemic stroke.

### 1.1. Stroke Epidemiology and Pathophysiology

Stroke risk factors are often multifactorial and complex but can be subdivided into nonmodifiable and modifiable. Nonmodifiable risk factors include age, sex, ethnicity, and genetics [5]. Modifiable risk factors include hypertension, hyperlipidemia, atrial fibrillation, cardiovascular disease, diabetes mellitus, and cigarette smoking [6,7,8,9,10]. Aggressive modifiable risk factor management has been shown to reduce stroke risk and is the mainstay of primary and secondary stroke prevention. Lowering blood pressure by 10 mmHg systolic or 5 mmHg diastolic was associated with a 41% reduction in stroke incidence [11]. In a review of 18 studies on hypertension and stroke risk in women, a 10 mmHg systolic increase was associated with a 38% increased risk of stroke and a 31% decreased risk of stroke with the use of just one antihypertensive medication [12]. Similarly, in a randomized controlled trial of patients treated with a high-intensity statin daily, there was a 16% relative risk reduction of fatal and nonfatal strokes [13]. A meta-analysis showed treatment of atrial fibrillation with warfarin reduced stroke by 62% [14]. A subsequent meta-analysis revealed that non-vitamin K oral anticoagulants were superior to warfarin for stroke prevention and systemic embolism [15].

AIS occurs due to an abrupt cessation of blood flow to the brain parenchyma via vaso-occlusive disease, leading to a lack of oxygenation and eventual cell death [16]. Large vessel occlusions (LVO), which are the prime focus of reperfusion therapy, can become occluded due to plaque rupture of pre-existing intracranial atherosclerosis, cardioembolic phenomenon, vascular dissection, or embolic stroke of an undetermined source. LVOs constitute 40% of AIS etiology and carry the worst prognosis with high morbidity and mortality [17,18,19]. Furthermore, infarcted tissue experiences acidosis, inflammation, excitotoxicity, free radical-mediated toxicity, cytokine-mediated cytotoxicity, complement activation, oxidative stress, breakdown of the blood–brain barrier, and infiltration of leukocytes [5]. This pro-inflammatory cellular cascade is the cornerstone of current investigations in neuroprotection after treatment of the occlusive etiology.

### 1.2. Penumbra

The discovery of the ischemic core versus penumbra paved the way for current stroke reperfusion therapies by identifying a new treatment target: salvageable ischemic tissue. Initial animal studies in the 1970s surfaced evidence of an irreversibly infarcted core surrounded by a halo of critically hypoperfused brain tissue with potential for recovery [20,21]. In addition to distance from the core, progressive tissue death was consequently determined to be time-dependent [22,23]. Over the next few decades, advances in AIS management have been focused on penumbra detection and reperfusion therapies aimed at parenchymal salvage.

### 1.3. Stroke and LVO Detection

First-line imaging for initial evaluation of stroke is typically non-contrast computer tomography head (NCCTH), which is highly sensitive for distinguishing hemorrhagic versus ischemic stroke. In AIS specifically, NCCTH can uncover LVO via hyperdense signs or early ischemic changes such as loss of gray–white matter differentiation or sulcal effacement. NCCTH is an optimal first-choice imaging modality due to its rapid acquisition while providing enough clinical information to screen for reperfusion candidacy [24]. Magnetic resonance imaging (MRI) with diffusion weighted imaging (DWI) is the gold standard of stroke delineation due to its high sensitivity, specificity, and accuracy [24,25,26]. 

Multimodal stroke protocols, including computer tomography angiography or magnetic resonance angiography, identify extra- and intracranial vaso-occlusive disease, which can also serve as indicators for reperfusion candidate selection. MRI DWI/perfusion weighted imaging (PWI) and cerebral computed tomography with perfusion (CTP) are the most widely utilized imaging modalities for penumbra detection. MRI PWI within 6 h of stroke onset has high sensitivity and specificity for salvageable penumbra [27,28]. Practical limitations such as extensive pre-screening, claustrophobia, and pacemaker contraindications make MRI less suitable for acute settings. In this regard, CTP delivers faster acquisition with the additional benefit of artificial intelligence processing software for LVO and penumbra detection. It offers high sensitivity in detecting core, however, has less specificity for differentiating core from penumbra [25,29,30]. Additionally, CTP is limited in differentiating other pathologies affecting cerebral blood flow, such as chronic strokes, mass lesions, and seizures from acute stroke, despite artificial intelligence software. Ultimately, selecting candidates for reperfusion therapy using core-penumbra mismatch is associated with favorable clinical outcomes [31]. 

## 2. Current Stroke Treatment

The natural course of LVO without revascularization results in severe disability (modified Rankin scale [mRS] 3–6) in 87–95% of patients and mortality in 78% of patients, indicating an imperative need for reperfusion therapies [32,33,34,35]. The two mainstays of AIS treatment are intravenous thrombolytics (IVT) and endovascular thrombectomy (EVT). Both techniques are rooted in recanalizing a LVO either by chemically dissolving or mechanically retrieving a clot, respectively. They can be used in tandem or in isolation, broadening the scope of treatment. The mantra “time is brain” is a cornerstone of reperfusion therapy and led to system-wide efforts to streamline administration of IVT and activation of the angiography suite. Early reperfusion therapy in the so-called “golden hour”, defined as the first 60 min after symptom onset, is thought to improve clinical outcomes due to greater tissue salvage with earlier recanalization [36,37,38,39].

### 2.1. Intravenous Thrombolysis 

Intravenous tissue plasminogen activator (tPA), or alteplase, was the first approved IVT for AIS treatment in 1996 based on the National Institute of Neurological Disorders study showing the benefit of tPA administered within 3 h of symptom onset as compared to placebo in regard to 24-hour and 90-day mRS. tPA was proven to improve clinical outcomes at 3 months, despite a 6% increased incidence of intracerebral hemorrhage (ICH) [40]. A subsequent trial in 2008, ECASS III, extended the narrow therapeutic window to 4.5 h but introduced stricter inclusion criteria regarding age, concurrent use of anticoagulants, history of prior stroke, and high symptom severity [41,42,43]. These contraindications were revised in later guidelines to suggest a more nuanced approach to determining eligibility [44]. More recently, tenecteplase (TNK) has begun to replace tPA as the new IVT after the Tenecteplase Versus Alteplase Before Endovascular Therapy for Ischemic Stroke (EXTEND-IA TNK) trial showed higher rates of reperfusion, better functional outcomes, and comparable rates of ICH [45]. In contrast to the two-step bolus and continuous infusion set up for tPA, TNK offers the appeal of a single-dose bolus administration.

Limitations of IVT include a narrow therapeutic window (3–4.5 h), prior history of ICH or risk of hemorrhagic transformation (HT), prior use of novel oral anticoagulants, and low recanalization rates in large arteries [46]. Despite practicing extended window tPA per American Heart Association (AHA) guidelines, about 75% of candidates were ineligible solely due to delay in presentation [42,47]. The risk of symptomatic ICH is low for both tPA and TNK at about 1% [45], but not negligible as all ICH rates are higher. Most notably, the low recanalization rate after IVT is likely the strongest indicator for needing additional interventions. A study of 60 patients by Christou et al. [48] reported a low complete recanalization rate of only about 32% after IV tPA. Another study of 300 patients reported an even lower early recanalization rate of 12% after IV tPA [49]. A meta-analysis by Rha et al. [50] found the recanalization rate with IVT was about 46% as compared to 84% with EVT.

### 2.2. Thrombectomy 

Endovascular therapy has transformed acute stroke treatment in fundamental ways. The first series of pivotal clinical trials describing the benefit of EVT for anterior circulation LVO were the Multicenter Randomized Clinical Trial of Endovascular Treatment for Acute Ischemic Stroke in the Netherlands (MR CLEAN), Endovascular Treatment for Small Core and Anterior Circulation Proximal Occlusion with Emphasis on Minimizing CT to Recanalization Times (ESCAPE), Solitaire with the Intention for Thrombectomy as Primary Endovascular Treatment (SWIFT PRIME), Extending the Time for Thrombolysis in Emergency Neurological Deficits—Intra-Arterial (EXTEND IA) and Randomized Trial of Revascularization with Solitaire FR Device versus Best Medical Therapy in the Treatment of Acute Stroke Due to Anterior Circulation Large Vessel Occlusion Presenting within Eight Hours of Symptom Onset (REVASCAT) conducted from 2010 to 2014 with a burst of results all published in 2015. The MR CLEAN trial revealed EVT within 6 h of symptom onset was effective and safe, as well as having higher rates of improved outcomes of mRS 0–2 as compared to IV tPA alone [51]. The ESCAPE trial was stopped early due to the efficacy of EVT, which showed improved functional outcomes and reduced mortality as compared to IV tPA alone [52]. The SWIFT PRIME trial was also stopped early due to the efficacy of stent retriever EVT within 6 h, showing improved 90-day mRS [53]. The EXTEND-IA trial used perfusion imaging guidance and was similarly stopped early due to the efficacy of stent retriever EVT based on improved reperfusion, early neurologic recovery, and better functional outcomes [54]. Lastly, the REVASCAT trial also showed the benefit of EVT within 8 h of symptom onset and better functional independence at 90 days [55]. A pivotal meta-analysis in 2016, HERMES, performed a pooled analysis of these five trials and ultimately showed improved functional outcome in patients treated with EVT within 12 h of symptom onset as compared to standard medical care, thereby paving the way for EVT to become a pillar of emergency revascularization for AIS [56].

Subsequent EVT trials focused on expanding the scope of EVT. The initial trials studied EVT in populations with symptom onset within 12 h, anterior circulation LVO, and small-sized core infarcts. In 2018, the EVT therapeutic window was extended to 24 h based on the results of the Diffusion Weighted Imaging or Computerized Tomography Perfusion Assessment With Clinical Mismatch in the Triage of Wake-Up and Late Presenting Strokes Undergoing Neurointervention (DAWN) and Endovascular Therapy Following Imaging Evaluation for Ischemic Stroke 3 (DEFUSE 3) trials [57,58]. Analysis of Pooled Data From Randomized Studies of Thrombectomy More Than 6 Hours After Last Known Well (AURORA), a meta-analysis of six late-window EVT trials, showed almost double the rate of 90-day mRS 0–2 in the EVT group compared to the control [59]. Despite the immense success of EVT in anterior circulation, EVT for basilar artery LVO was not demonstrated to be superior to medical management until 2022 in the Endovascular Treatment for Acute Basilar Artery Occlusion (ATTENTION) and Basilar Artery Occlusion Chinese Endovascular (BAOCHE) trials studying 12 h and 24 h windows, respectively [60,61]. Multiple meta-analyses confirmed that EVT for BAO was associated with lower mortality and better outcomes than medical management in patients with high symptom severity [62,63,64]. Most recently, a series of clinical trials, including the Randomized Controlled Trial of Endovascular Therapy for Acute Large Vessel Occlusion With Large Ischemic Core (RESCUE Japan LIMIT), the Trial of Endovascular Therapy for Acute Ischemic Stroke with Large Infarct (ANGEL-ASPECT), and the Trial of Endovascular Thrombectomy for Large Ischemic Strokes (SELECT2), have further broadened the scope of EVT to include large core infarcts by showing the benefit of EVT over medical management alone, but at risk of higher rates of ICH [65,66,67].

A summary of the above-mentioned studies can be found in Table 1. They show high recanalization rates, measured using the Modified Thrombolysis in Cerebral Infarction (mTICI) score, but suboptimal functional outcomes. The International Acute Ischemic Stroke Registry with the Penumbra System Aspiration Including the 3D Revascularization Device (COMPLETE) study showed about an 87% success rate of mTICI 2b-3 revascularization [68]. However, a retrospective study showed lower rates of functional independence at 90 days with mTICI 2b revascularization as compared to TICI 2c-3 [69]. Furthermore, in the CT for Late Endovascular Reperfusion (CLEAR) study, about 50% of patients had mRS 3–6 at 90 days despite successful late-window mTICI 2c-3 revascularization [70]. This gap between the outcomes of recanalization and symptomatic improvement suggests a need for adjunctive therapies in post-thrombectomy care to further optimize functional outcomes.

## 3. Neuroprotection

Stroke preclinical models showed recanalization alone does not halt infarct growth, likely due to ischemia-reperfusion injury as well as inflammatory microthrombus formation [71]. EVT can lead to high production of reactive species secondary to reperfusion, further damaging ischemic tissue, which is inherently vulnerable to oxidative stress [72]. A wide range of neuroprotective therapies have been under investigation to bridge this discrepancy in continued infarct growth. Early preclinical models over the last 25 years have shown poor translational promise as studies sifted through countless potential targets. Major pitfalls of animal models included difficulties in replicating the heterogeneity of stroke, modeling comorbidities, and utilizing aging populations [73]. The choice of animal model used to induce ischemia also introduced significant variability in insult type and likely contributed to translational failure [74]. These initial efforts were primarily focused on neuron rescue and excitotoxicity suppression [75]. An extensive preclinical study on meta-analysis of cellular agents as well as experimental combination therapy with magnesium, melatonin, and minocycline failed to show the benefit of this trio in reducing infarct volume or mortality in rats [76]. However, with more insight into ischemia-reperfusion injury, recent preclinical testing has shifted towards preventing oxidative stress through upregulation of antioxidants and downstream effectors, with some positive results [77,78,79]. Just as recanalization alone is insufficient, neuroprotection alone will also not suffice. The future of stroke care will likely require a hand-in-hand approach with tailored combination therapies of reperfusion and neuroprotection. An overview of promising neuroprotective therapies can be found in Table 2.

### 3.1. Uric Acid

Uric acid is a product of purine metabolism whose mechanism of neuroprotection is not fully elucidated, but the agent is well known for its antioxidant property. Uric acid scavenges reactive nitrogen and oxygen species, suppresses the Fenton reaction, and limits free radical damage to DNA, thus preventing oxidative stress [80]. Another proposed neuroprotective pathway involves uric acid’s ability to downregulate vascular endothelial growth factor (VEGF), particularly VEGF-A, via upregulation of Krüppel-like factor 2, a transcription factor that regulates endothelial cell growth, differentiation, and activation [81]. VEGF-A promotes angiogenesis, which is associated with neuroprotective qualities. One of the body’s responses to ischemic stroke is upregulation of VEGF-A, which contributes to exaggerated angiogenesis, leading to disruption of blood–brain barrier (BBB) integrity, edema, hemorrhage, and brain damage [81]. In 2020, a retrospective analysis was conducted on 247 patients who underwent EVT and experienced HT within 72 h [82]. Patients with HT had significantly lower uric acid levels compared to those without HT (322.60 ± 94.49 vs. 350.25 ± 99.28 μmol/L, *p* = 0.032) [82]. Another prospective cohort study evaluated uric acid levels within 24 h of EVT and their correlation with an excellent 90-day functional outcome, defined by mRS 0–1 [83]. Multivariate analysis showed that a higher uric acid level was significantly associated with an excellent functional outcome (*p* = 0.018). In the Efficacy Study of Combined Treatment With Uric Acid and r-tPA in Acute Ischemic Stroke (URICO-ICTUS) trial, patients were randomized to receive either intravenous 1000 mg uric acid or placebo along with IVT. This was followed by thrombectomy in a subgroup of 45 patients. The primary outcome, good functional outcome defined by mRS 0–2, was observed in 67% of patients treated with uric acid and 48% treated with placebo (adjusted odds ratio 6.12, 95% CI 1.08–34.56) when receiving both IVT and EVT [84]. Support for these results requires further investigation in a larger clinical trial, and in addition, it can focus more on EVT. 

### 3.2. Activated Protein C: 3K3A-APC

Activated protein C (APC) is a serine protease with independent cytoprotective effects mediated by activation of protease activated receptor 1 (PAR-1) and anticoagulant effects mediated by inactivation of clotting factors Va and VIIIa [85]. APC cleaves PAR-1, initiating the β-arrestin-2 pathway, with the downstream effects of promoting anti-inflammatory and anti-apoptotic activities, BBB stabilization, neurogenesis, and neovascularization in ischemic stroke recovery [86]. APC’s anticoagulation properties are less desirable due to concerns about post-perfusion hemorrhage [87]. In order to decrease bleeding risk while preserving its effect on PAR-1, a variant of APC was engineered by ZZ Biotech, resulting in the molecule 3K3A-APC, which has greater than 90% of the anticoagulant activity removed. The NeuroNEXT trial, Safety Evaluation of 3K3A-APC in Ischemic Stroke, which investigated 3K3A-APC’s cytoprotective properties in protecting ischemic brain tissue while decreasing treatment-related bleeding, recently concluded phase II of its clinical trials. One hundred and ten patients were randomized to one of four doses, 120, 240, 360, and 540 μg/kg, or placebo after receiving IVT, EVT, or both. The primary outcome of this trial was to establish a maximum tolerated dose (MTD), defined as the highest study dose with an estimated dose-limiting toxicity rate of 10% or less. Dose-limiting toxicity for the highest dose, with an estimated toxicity rate of 7%, was not statistically different from placebo. In exploratory analyses, 3K3A-APC reduced ICH rates by 67.4% compared to placebo, 86.5% (*p* = 0.046). Total hemorrhage volume was also reduced from an average of 2.1 ± 5.8 mL in the placebo to 0.8 ± 2.1 mL in the combined treatment arms but was not statistically significant [87]. Rilonacept Inhibition of Interleukin-1 Alpha and Beta for Recurrent Pericarditis: A Pivotal Symptomatology and Outcomes Study (RHAPSODY) II is a phase III clinical trial that is planned and will further elucidate the safety and efficacy of 3K3A-APC.

### 3.3. Nerinetide

Nerinetide is a synthetic peptide designed to disrupt postsynaptic density protein (PSD-95), a scaffolding protein that interacts with neurotoxic signaling agents, by inhibiting PSD-95′s interactions with N-methyl-D-aspartate (NMDA) glutamate receptors and negating downstream excitotoxicity effects [88]. The phase III trial, Efficacy and Safety of Nerinetide for the Treatment of Acute Ischaemic Stroke (ESCAPE-NA1), with collaborative efforts from eight different countries, evaluated the safety and efficacy of nerinetide [89]. The trial took approximately 2 years, in which 1105 patients were randomized to receive nerinetide or placebo after EVT; select patients also received IVT before or during EVT, and indications for administering alteplase were based on the national or regional guidelines of each institution. The primary outcome was a favorable functional outcome after 90 days, defined as mRS 0–2. Of the nerinetide group, 61.4% of patients achieved mRS 0–2, while in the placebo group, 59.2% achieved mRS 0–2 at 90 days (adjusted risk ratio: 1.04, 95% CI 0.96–1.14; *p* = 0.35). Adverse outcomes such as symptomatic ICH, recurrent or new-onset stroke, and progression of stroke were equal in both groups. The results suggested no significant difference in the improvement of outcomes between nerinetide and placebo. However, there was concern for effect modification resulting in inhibition of the treatment effect in patients receiving tPA. The thrombolytic agent was given to 60.1% of the patients in the nerinetide group. Upon pharmacokinetic analysis, patients who received tPA had subtherapeutic plasma concentrations of nerinetide compared to those who received only EVT. These subtherapeutic levels were thought to be the consequence of the cleavage of nerinetides’ amino acid sequences by plasmin, formed from alteplase-activated plasminogen. Consequently, they found a significant difference only when looking at the stratum of patients that were not given tPA, with 59.3% of those receiving nerinetide achieving favorable functional outcomes compared to 49.8% receiving placebo (adjusted RR 1.18, 95% CI 1.01–1.38). This led to a 7.5% absolute risk reduction in mortality at 90 days and a halving of the hazard of death. With the drug-drug interaction considered, the next phase of the nerinetide trial, ESCAPE-NEXT, is already in progress with the design to exclude thrombolysis via tPA [89].

### 3.4. Otaplimastat 

Otaplimastat (SP-8203) is a quinazoline molecule with cytoprotective properties. It upregulates the inhibitor of metalloproteinase-1, thus inhibiting matrix metalloproteinase (MMP), an enzyme associated with BBB breakdown leading to edema [90]. Otaplimastat is also shown to inhibit NMDA receptor-mediated neuronal calcium influx and reduce the production of reactive oxygen species [91]. The Safety and Efficacy of Otaplimastat in Patients with Acute Ischemic Stroke Requiring tPA (SAFE-TPA), a phase II clinical trial, randomized 69 patients into three groups: otaplimastat 40 mg, otaplimastat 80 mg, or placebo, after administering IVT along with EVT if LVO was detected on MRI and MRA [90]. The primary endpoint was the occurrence of parenchymal hematoma on day 1. Secondary endpoints included mRS distribution at 90 days and serious adverse events (SAEs). The secondary safety endpoint included the incidence rate of symptomatic ICH within 5 days. There was no significant difference between the incidence of parenchymal hematoma: 0 of 22 with the placebo, 0 of 22 with otaplimastat 40 mg, and 1 of 21 with otaplimastat 80 mg. Fisher’s exact test suggests a significantly different distribution of mRS scores with otaplimastat 40 mg versus placebo at 90 days (*p* = 0.026), but not with otaplimastat 80 mg (*p* = 0.502). However, the true trends of otaplimastat’s efficacy were limited by the small sample size (adjusted odds ratio 3.2, 95% CI 0.9–10.9, *p* = 0.068). The incidence of SAEs, such as myocardial infarctions or cerebral infarctions, was similar between each group: 3% for placebo, 17% for otaplimastat 40 mg, and 14% for otaplimastat 80 mg. Secondary safety endpoints included the incidence rate of symptomatic ICH within 5 days, which none of the three groups experienced. The study provided evidence that giving otaplimastat to patients receiving IVT is generally safe. The efficacy of this drug cannot be firmly established due to the small size of this trial [90]. Further studies are needed to examine the efficacy of EVT alone. 

### 3.5. Imatinib 

Imatinib, more widely recognized as a chemotherapy agent, has been shown to have neuroprotective properties as well. The proposed mechanism is its inhibitory effect on signaling of platelet-derived growth factor alpha (PDGF-alpha) receptors on perivascular astrocytes, which affects the opening of the BBB; downstream effects seen in experimental models showed a decrease in BBB permeability and a reduction in infarct size with associated lower risks of HT and cerebral edema [92]. I-STROKE, a phase II trial, randomized 60 patients into four groups: three active treatments with differing doses and one placebo, after receiving IVT along with EVT if LVO was detected on imaging. The primary outcome was any adverse event; secondary outcomes were HT, neurological severity on the National Institutes of Health Stroke Scale (NIHSS) at 7 days and at 3 months, and functional outcomes based on mRS. A total of four serious adverse events were reported: three deaths that investigators thought were unrelated to study treatment and one femoral artery puncture site infection in the control group. No HT (0 of 5) occurred when high-dose imatinib was initiated within 5 h after stroke onset. HT occurred in 4/9 patients in the medium dose group and 4/8 patients in the small dose group when imatinib was initiated within 5 h after stroke onset; in the controlled group, 7/18 patients experienced HT. There were significant improvements in the mean NIHSS scores in patients treated with high-dose imatinib compared to controls before adjustment (*p* = 0.037) and after adjustment for thrombectomy (*p* = 0.012). In terms of functional independence (mRS 0–2), the high-dose group saw an 18% absolute increase compared to control but not at statistical significance [92]. A larger Phase III clinical trial is underway and will hopefully confirm the positive results of this Phase II trial.

### 3.6. Verapamil

Verapamil, an L-type calcium channel blocker, was shown to have neuroprotective qualities during in vivo experiments. In mouse models, administration of intra-arterial verapamil following recanalization of middle cerebral artery occlusion reduced infarct volume, improved immunohistochemical markers of neuron preservation, and reduced astrogliosis. Though the mechanism remains to be elucidated, it is hypothesized to be secondary to a reduction in excitotoxic damage from calcium-mediated apoptosis [93]. Superselective Administration of Verapamil during Recanalization in Acute Ischemic Stroke (SAVER-I), a phase I trial, enrolled 11 of 104 patients who met inclusion criteria to undergo EVT immediately, followed by intra-arterial administration of 10 mg of verapamil. All 11 subjects were successfully treated, and none met the primary safety endpoint, which was significant ICH. The trial demonstrates the safety and potential neuroprotective efficacy of direct verapamil administration after reperfusion from EVT. The obvious limitations, such as the lack of a control group and the power of the study, should be addressed in further trials [93].

### 3.7. Butylphthalide

Butylphthalide (NBP) is a compound that can be extracted from the seeds of *Apium graveolens* (Chinese celery). The active form, dl-3-N-NBP, has been shown to reduce cerebral ischemic damage based on animal experiments [94]. The underlying mechanisms include promotion of microcirculation, protection of the BBB, reduction of mitochondrial dysfunction, and prevention of post-stroke inflammation and cerebral edema [94]. Butylphthalide for Acute Ischemic Stroke Patients Receiving Intravenous Thrombolysis or Endovascular Treatment (BAST), a phase III trial, randomized 1216 of 1236 patients to receive NBP or placebo along with IVT, EVT, or both [95]. The primary efficacy outcome was the proportion of patients with a favorable outcome at 90 days, defined by mRS 0–2. In the NBP group, 56.7% of patients achieved favorable functional outcomes, a significant difference compared to 44.0% in the placebo group (OR 1.70, 95% CI 1.35–2.14, *p* < 0.001). Primary safety outcomes were SAE within 90 days, which included prolonged hospital time, permanent damage to the body or organ, and death. Of the NBP group, 61 (10.0%) experienced SAE, compared to 73 (12.0%) in the placebo group [95]. Overall, the outcomes of this trial support NBP’s efficacy in improving 90-day functional outcomes. Future studies should focus on thrombectomy alone, as only a small percentage of the participants received EVT.

### 3.8. Edaravone Dexborneol 

Edaravone is an antioxidant that improves ischemic stroke outcomes through scavenging hydroxyl-, peroxyl-, and superoxide-free radicals, relieving cerebral edema, and inhibiting delayed neuronal death [96]. Moreover, (+)-bornel is a naturally occurring compound that inhibits the production of inflammatory factors and preserves brain function in preclinical investigations. The novel drug edaravone dexborneol, composed of edaravone and (+)-bornel, has shown synergistic effects in neuroprotection. Treatment of Acute Ischemic Stroke with Edaravone Dexborneol (TASTE), a phase III trial, investigated edaravone dexborneol versus edaravone on 90-day functional outcomes, defined by mRS less than or equal to 1, in patients with AIS. The study randomized 1200 patients to receive edaravone, dexborneol, or edaravone along with EVT. There were 393 (67.18%) patients in the edaravone dexborneol group and 342 (58.97%) patients in the edaravone group with a mRS score less than or equal to 1 (OR 1.42, 95% CI 1.12–1.81, *p* = 0.004). Analysis of safety outcomes indicated that the two treatment groups had similar incidences of serious adverse events such as cerebral infarction or lung infection: 54 (9.02%) with edaravone dexborneol versus 47 (7.90%) with edaravone alone [96]. Results suggest 90-day good functional outcomes favored the edaravone dexborneol group. A limitation in this study was the lack of a placebo group, which will hopefully be addressed in future trials.

### 3.9. Nelonemdaz (Neu2000)

Nelonemdaz, previously known as Neu2000, is a synthetic derivative of sulfasalazine and aspirin that acts as an NMDA receptor subtype 2B selective antagonist. In contrast to the 2A subunit, the 2B subunit is mostly involved in pro-death signaling [97]. Glutamate is released and accumulates during ischemia, leading to the activation of NMDA receptors and an influx of excess calcium, resulting in the activation of cytotoxic proteins and eventual cell death [98]. In addition, nelonemdaz acts as a free radical scavenger to mitigate the oxidative damage that results not only downstream of NMDA activation but also following reperfusion [98,99]. By targeting two different pathways of nerve cell death following ischemic injury, there is potential for enhanced neuroprotection compared to monotherapy alone.

Following preclinical studies that showed efficacy against middle cerebral artery occlusion in rats and phase I studies that showed safety in total doses up to 6000 mg in healthy volunteers, Hong et al. [100] conducted the Safety and Optimal Neuroprotection of Neu2000 in Acute Ischemic Stroke With Recanalization (SONIC) trial, a phase II randomized controlled trial (RCT) in 208 patients with AIS due to anterior circulation LVO receiving EVT within eight hours of symptom onset [98,101]. Nelonemdaz was administered in either low-dose or high-dose groups. An initial larger infusion (500 or 750 mg) before thrombus retrieval as well as multiple smaller doses (250 or 500 mg q12h over five days) allow targeting of both the initial glutamate-mediated excitotoxicity and the subsequent free radical-mediated oxidative damage. There were no significant adverse events. However, there was no statistically significant difference between these two groups or placebo in the proportion of patients achieving mRS 0–2 at 12 weeks. Nevertheless, its safety and favorable trend in mRS are promising [100]. As a result, it is being further investigated in a current phase III trial called Rescue on Reperfusion Damage in Cerebral Infarction by Nelonemdaz (RODIN), with a look at the primary outcome of mRS at 90 days [97].

### 3.10. ApTOLL

ApTOLL is a single-stranded DNA aptamer that acts as an antagonist of toll-like receptor 4 (TLR4), a receptor that is expressed in microglia and astrocytes and is involved in innate immunity. It recognizes not only exogenous ligands from infectious agents, such as pathogen-associated molecular patterns, but also endogenous ligands, such as damage-associated molecular patterns, that are released as a result of cellular damage in a variety of conditions. Its activation leads to the recruitment of adaptive proteins and the activation of nuclear factor κ-B, resulting in the expression of proinflammatory mediators [102]. In AIS, TLR4 was associated with poor outcomes, suggesting that inflammation further contributes to the damage [102]. This is supported by preclinical studies. Mice deficient in TLR4 were found to have a reduced expression of proinflammatory mediators, such as inducible nitric oxide synthase, cyclooxygenase-2, interferon regulatory factor-1, and MMP9 [103]. Furthermore, blocking TLR4 in wild-type mice had a protective effect against brain injury in an experimental stroke [104].

A phase I study determined the half-life to be 9.3 h, which would allow ApTOLL to be active during the acute inflammatory process and inactive during the later stages of tissue repair [105]. Hernández-Jiménez et al. [106] then conducted a phase Ib/IIa study called APRIL in patients with AIS due to anterior circulation large vessel occlusion receiving EVT within six hours after symptom onset. The study was divided into two parts. The first part was an ascending dose study in 32 patients that collected safety data. After using this information to determine the appropriate doses, the second part randomized another 119 patients into three arms (0.05 or 0.2 mg/kg ApTOLL or placebo), with the trial drug being administered as an infusion as soon as possible after randomization and before initiation of EVT. They found that ApTOLL, in combination with EVT, was safe and well-tolerated. While the study was not sufficiently powered to definitively determine efficacy, patients receiving the 0.2 mg/kg dose had reduced mortality at 90 days, warranting further phase III study [106].

### 3.11. Regional Hypothermia

Therapeutic regional hypothermia (TRH) consists of specifically targeting the brain for cooling while keeping the remainder of the body at a higher temperature. Neuroprotection is presumed to be achieved through a reduction in basal metabolic rate. Targeted regional hypothermia provides the benefit of faster achievement of target temperatures while attempting to avoid side effects associated with systemic cooling. TRH can be achieved through helmets, intranasal sprays, and direct cooling via large arteries like the carotid arteries. A pilot study investigated the safety and feasibility of intraarterial cold saline infusion in combination with EVT [107,108]. In this study, 26 patients with LVO had cold saline directly infused into either the internal carotid artery or vertebral artery both prior to and after EVT. The temperature of the ischemic tissue was reduced by about 2 degrees Celsius, but the overall body temperature was only mildly reduced (a maximum of 0.3 degrees Celsius). There were no significant adverse events. Another RCT comparing targeted hypothermia to normothermia in patients with middle cerebral artery occlusion showed mean infarct volumes and neurological deficits were significantly lower in the hypothermia group [109]. Studies have shown TRH to be both safe and effective. However, larger clinical trials need to be conducted to show consistent results before consideration can be given to the development of a universal TRH protocol and clinical application.

### 3.12. Remote Ischemic Conditioning

Remote ischemic conditioning (RIC) is the process of strengthening endogenous mechanisms to build tolerance against ischemic damage. In this model, ischemia is induced in tissue just below the threshold of damage to build tolerance against more severe ischemia. A meta-analysis by Mollet et al. [110] has summarized remote ischemic conditioning experimental models and mechanisms of action. When ischemic stimuli are applied to a limb, there is inter-organ communication between limb and brain via activation of hypoxia-inducible factor-alpha, which leads to downstream upregulation of over 200 genes responsible for the adaptation to hypoxia. Although RIC was shown to be safe, there was no proven benefit [110]. More recently, the REmote iSchemic conditioning in patients with acute STroke (RESIST) trial used an inflatable cuff to apply pressure to one limb. This trial failed to show functional improvement, measured as mRS at 90 days, in those who received RIC compared to the control group [111]. However, a similarly designed study in China (RICAMIS) showed that the likelihood of excellent functional outcome at 90 days, defined as a mRS score of 0–1, was more likely in the group treated with RIC [112]. The RICAMIS study applied cuff pressure to two limbs for 10–14 days, whereas in the RESIST trial, the duration of therapy was 7 days and cuff pressure was applied to one limb. Given the lack of consistent findings, replication is necessary, perhaps with other modalities of ischemia induction, in order to better clarify the efficacy of RIC in neuroprotection after an ischemic stroke.

### 3.13. Normobaric Oxygen

Lack of oxygen supply is a critical pathophysiology of ischemic stroke and, therefore, another area of potential intervention for neuroprotection. Normobaric oxygen (NBO) therapy aims to increase the partial pressure of oxygen, increase oxygen supply to the penumbra, and thereby reduce ischemic damage [113]. NBO is defined as breathing oxygen between 21 and 100%. The oxygen saturation of a healthy individual at sea level is at least 95%, so supplemental oxygen cannot further increase the amount of oxygen bound to hemoglobin. Therefore, NBO aims to increase the partial pressure of oxygen in the blood and the partial pressure of oxygen in brain tissue. The hope is to increase the oxygen level of the penumbra, thereby reducing infarct volume and deficits from stroke [113].

Studies in rat models showed smaller infarct sizes in NBO-treated rats [114]. However, meta-analysis reveals that subsequent human model studies failed to show benefit with NBO treatment, which could be due to the major limitation of including study participants who did not receive recanalization therapy [114,115]. With continued efforts, a recent RCT conducted at Beijing Luhe Hospital at Capital Medical University investigated the benefit of high-flow NBO therapy after EVT of anterior circulation strokes. A total of 180 patients, aged 18–80, with NIHSS 6–25 and anterior LVO and less than 6 h from stroke onset to EVT, were enrolled in this study. Of the 180 participants, 91 were enrolled in the NBO group and received oxygen via nasal cannula during thrombectomy and then supplemental high-flow NBO treatment via Venturi mask for 6 additional hours. This study showed a significant reduction in 90-day mortality (13.86%) and reduced infarct volume in those who received NBO therapy for 6 h after recanalization [116].

### 3.14. Nuclear Factor Erythroid 2-Related Factor 2

Nuclear factor erythroid 2-related factor 2 (Nrf2) is a transcription factor that regulates the activation of over 250 cytoprotective genes involved in the production of antioxidants, anti-apoptotic proteins, and proteasome proteins [117,118,119,120]. Nrf2 signaling pathways are also implicated in redox regulation, suppression of inflammatory genes, angiogenesis, and cell proliferation and migration [121,122,123,124,125]. In particular, Nrf2 downstream proteins have been shown to neutralize reactive oxygen species byproducts of ischemic reperfusion injury by recruiting enzymes such as glutathione and superoxide dismutase [126,127]. Given its essential role in maintaining homeostasis after ischemic injury, several studies exploring the role of Nrf2 in neuroprotection after AIS are underway. Ischemic stroke models in rodents have revealed neuroprotective effects of exogenously induced Nrf2 activation with red ginseng, apelin 13, astragaloside IV, rehmannioside A, loureirin C, and Srs11-92, among many others. In addition to inhibiting ferroptosis, decreasing neuroinflammation, and reducing oxidative stress, the neuroprotective effects of these agents include reduced brain infarct size, improved neurological outcomes, and improved cognition [128,129,130,131,132,133,134]. Ischemic preconditioning with icariside II, a naturally occurring Nrf2 activator, exerted cytoprotective effects on astrocytes and inhibited ferroptosis [135]. More notably, ischemic preconditioning with the BBB permeable Nrf2 activator 2-cyano-3,12-dioxo-oleana-1,9(11)-dien-28-trifluoethyl amide was shown to reduce sensorimotor deficits, post-stroke cognitive impairments, and brain tissue loss in mice [136]. However, dimethyl fumarate for the treatment of multiple sclerosis is currently the only clinical therapeutic agent utilizing Nrf2 activation. With countless emerging studies on Nrf2 in AIS models, there is great promise for the development of additional agents.

## 4. Anesthetic Management for Thrombectomy

### 4.1. General Anesthesia versus Sedation

Anesthesiologists play an essential role in the management of patients undergoing EVT for AIS caused by LVO. Their goal is to facilitate recanalization as quickly as possible while maintaining perfusion to the brain to prevent additional ischemic injury [137]. Some considerations include the type of anesthesia and hemodynamic management, which have an impact on functional outcomes. However, the optimal anesthetic strategy remains a topic of debate, with each having its own advantages and disadvantages. Compared to general anesthesia, sedation allows for the assessment of neurologic status during the procedure. However, an unprotected airway poses a potential risk for pulmonary aspiration and hypoventilation, and conversion to general anesthesia may be required in cases of clinical deterioration. Furthermore, while the procedure is not very stimulating and requires minimal analgesia, clot extraction could lead to some discomfort. As a result, general anesthesia may potentially improve success rates by preventing patient movement and improving imaging conditions. Nevertheless, the induction of general anesthesia may lead to a delay in the start time of the procedure and can lead to hemodynamic instability [138]. 

While earlier retrospective and observational studies showed that general anesthesia was associated with worse outcomes compared to sedation, more recent RCTs suggest this may not be the case. The Sedation vs. Intubation for Endovascular Stroke Treatment (SIESTA) study found no significant difference in early neurological improvement on NIHSS after 24 h, but a higher percentage of those receiving general anesthesia were functionally independent (mRS score 0–2) after 3 months (37% vs. 18.2%) [139]. The Anesthesia During Stroke (AnStroke) trial found no significant difference in mRS scores at 3 months [140]. Finally, the General or Local Anesthesia in Intra Arterial Therapy (GOLIATH) trial found smaller, but not statistically significant, infarct growth in those receiving general anesthesia with an improvement in mRS scores at 90 days [141].

A meta-analysis of 19 observational studies and these 3 RCTs, including 4716 patients, found that patients receiving general anesthesia had higher odds of mortality and lower odds of a good functional outcome compared to patients receiving either local anesthesia or sedation [142]. This was further supported by an updated meta-analysis of 13 non-randomized studies and 3 RCTs, including 5836 patients [143]. Conversely, when including only the three RCTs, which had a total of 368 patients, general anesthesia was associated with less disability at 3 months [144]. Reasons for this discrepancy could be due to several factors, such as selection bias in retrospective and observational studies and the possibility that patients with more severe symptoms are more likely to receive general anesthesia. The RCTs had standardized protocols for the administration of anesthesia and hemodynamic management but were limited to a single center and small sample sizes.

Maurice et al. [145] addressed these limitations with the General Anesthesia versus Sedation for Acute Stroke Treatment (GASS) trial, which was multicenter and randomized 351 patients receiving endovascular therapy into either general anesthesia or conscious sedation groups. Similar to the previous three studies, they implemented protocols for intraoperative hemodynamic control with a goal of systolic blood pressure (SBP) between 140 and 185 mmHg and diastolic blood pressure less than 110 mmHg. They found no difference in functional outcomes, with 36% of patients in the conscious sedation group and 40% of patients in the general anesthesia group achieving the primary outcome of a mRS score less than or equal to 2 at 3 months. Although those receiving general anesthesia did have more episodes of hypotension or hypertension, the cumulative duration was similar between the groups. Furthermore, while general anesthesia was associated with a longer time from onset and arrival to groin puncture (additional 19 and 9 min, respectively), there was increased success (85% vs. 75%) and a similar time to recanalization [145]. This suggests that either option is reasonable if hemodynamics are well controlled. 

Consistent with these findings, the 2019 guidelines from the American Heart Association/American Stroke Association (AHA/ASA) recommend that the anesthetic technique be chosen on an individual basis, considering patient risk factors and clinical characteristics [44]. This recommendation is similar to the expert consensus statement from the Society for Neuroscience in Anesthesiology and Critical Care (SNACC) [146]. Further research continues with the multicenter RCT Sedation versus General Anesthesia for Endovascular Therapy in Acute Ischemic Stroke (SEGA), which aims to compare functional outcomes after successful endovascular therapy. The primary outcome will be the mRS score at 90 days, and results have yet to be released [138]. Finally, the neuroprotective properties of different types of anesthetic agents may also be an avenue for future research.

### 4.2. Perioperative Blood Pressure Management

Hemodynamic management, especially blood pressure control, is a critical element in maximizing functional outcomes in patients with AIS. The presenting blood pressure in patients with AIS plays a complex role in the patient’s outcome. The AHA/ASA guidelines recommend that patients treated with tPA maintain blood pressure less than 185/110 mmHg and patients not treated with tPA maintain blood pressure less than 220/120 mmHg [44]. Studies have shown a U-shaped relationship between presenting blood pressure and outcomes, with high or low blood pressures correlating to worse outcomes [147,148,149]. Furthermore, higher blood pressure variability (BPV) after an ischemic stroke correlates to worse functional outcomes and death [150]. In patients undergoing EVT, optimal blood pressure parameters have not been established given the multiple factors that influence the effects of blood pressure on outcomes in these patients, such as baseline blood pressure, size of the penumbra, degree of collateral blood flow, the risk of reperfusion injury and hemorrhage, etiology of the stroke, etc.

High blood pressure, often SBP greater than 160 mmHg [151], is a typical presenting symptom of AIS and is attributed to the cerebral ischemic response, which evokes autonomic nervous system activation [152]. Blood pressure then decreases during the next seven days [147,153]. Looking at the data of 17,398 patients from the International Stroke Trial (IST), Leonardi-Bee et al. [147] found that 81.6% had an SBP greater than 140 mmHg (mean SBP of 160.1 mmHg) at the time of enrollment within 48 h of stroke onset. There was a U-shaped relationship, with baseline SBP 140 to 179 mmHg being associated with the lowest frequency of poor outcomes. The nadir was around 150 mmHg, with the risk of early death increasing by 17.9% for every 10 mmHg below and 3.8% for every 10 mmHg above [147]. Other studies have also found similar U-shaped relationships for presenting blood pressure and outcomes following AIS. Castillo et al. [149] found SBP 180 mmHg to be optimal, whereas Vemmos et al. [148] determined 130 mmHg as the optimal SBP. Recanalization status and cerebral autoregulation affect blood pressure parameters in AIS patients and contribute to this U-shaped curve [154]. Hypertension may lead to cerebral edema, reperfusion injury, and cerebral hemorrhage in recanalized patients.

Blood pressure parameters in AIS patients undergoing EVT differ pre-, intra-, and post-procedurally. In endovascular trials, pre-procedural blood pressure parameters followed AHA/ASA guidelines of blood pressure < 180/105 mmHg since these trials included patients eligible for intravenous t-PA [44]. The MR CLEAN trial showed that EVT was beneficial regardless of pre-procedural blood pressure, but a mean SBP of 120 mmHg correlated to more favorable outcomes. Poor functional outcomes, again, were correlated with low and high baseline SBPs [155]. In terms of intra-procedural blood pressure, endovascular trials did not show evidence to support specific blood pressure parameters during EVT. Most endovascular trials did not include intra-procedural blood pressure management protocols. The ESCAPE trial proposed the most specific intra-procedural blood pressure management protocol, specifying the maintenance of SBP greater than or equal to 150 mmHg prior to recanalization in order to promote collateral flow [52]. Intra-procedural blood pressure decreases of mean arterial pressure (MAP) less than 40% from baseline were associated with poorer outcomes and a greater infarct increase [156,157]. Analysis of blood pressure and outcome data from three randomized trials comparing general anesthesia with conscious sedation (GOLIATH, SIESTA, and AnStroke) demonstrated that intra-procedural MAP less than 70 mmHg for more than 10 min correlated with higher mRS at 90 days [158]. These three trials also demonstrated that a MAP greater than 90 mmHg for more than 45 min correlated to worse outcomes. Chen et al. [159] showed poorer functional outcomes in patients with intra-procedural SBP greater than 163 mmHg and MAP greater than 117 mmHg pre-recanalization. In a retrospective review, John et al. [160] reported a mean maximum intraprocedural SBP of 180.9 mmHg as an independent predictor of poor outcome. Overall, based on limited data and a lack of standardized protocols for intraprocedural blood pressure parameters in trials, generalizations advocating for specific blood pressure values during EVT are not broadly supported by the current literature. However, some trial evidence does point to intraprocedural hypotension correlating with worse outcomes. 

Blood pressure management post-thrombectomy in patients with AIS typically focuses on normotension following successful reperfusion and the avoidance of hypertension. Hypertension following successful reperfusion can lead to reperfusion injury and hemorrhagic transformation. Mistry et al. [161] showed that maximum blood pressure within the first 24 h after thrombectomy was independently associated with worse outcomes at 3 months and HT at 48 h in patients with anterior circulation LVO. Hemorrhages occurred at lower SBPs in patients who had successful reperfusion, supporting the theory that HT is linked to reperfusion injury [161]. Numerous observational studies have reported that high blood pressure and greater BPV post-recanalization are linked to worse functional outcomes [162,163]. The REVASCAT study targeted a blood pressure of less than 160/90 mmHg for patients with a TICI 2b flow or greater [55]. The Blood Pressure after Endovascular Therapy for Ischemic Stroke Trial (BEST), a prospective, multi-center cohort study, demonstrated that a maximum SBP > 158 mmHg was associated with a worse outcome in patients post-EVT [164]. Similarly, higher BPV in the first 24 h after thrombectomy correlated to worse outcomes at 90 days in a substudy of the BEST trial [165]. Current AHA/ASA guidelines recommend a blood pressure target of ≤180/105 mmHg during and for 24 h following EVT (IIa recommendation) [44]. 

Considering these findings, further research is needed to establish blood pressure parameters pre-, intra-, and post-thrombectomy. The AHA/ASA guidelines recommend a blood pressure < 185/100 mmHg in patients receiving intravenous thrombolysis in order to lower the risk of cerebral hemorrhage. The guidelines recommend blood pressure up to 220/120 mmHg in patients not receiving thrombolysis [44]. Specific intra-procedural blood pressure parameters for patients with AIS undergoing EVT remain uncertain. General recommendations for blood pressure management during EVT include the avoidance of hypotension (target SBP > 140 mmHg or MAP > 70 mmHg) until reperfusion is established. Patients with very high SBP prior to reperfusion appear to have worse outcomes, but data remain limited. After recanalization, the avoidance of hypertension (target SBP < 140 mmHg) is paramount in order to decrease the risk of reperfusion injury and HT [166]. Most importantly, understanding the limitations of these guidelines is essential in the provision of care to maximize functional outcomes in AIS. The hemodynamic parameters should be tailored to each patient based on the baseline blood pressure, collateral perfusion, degree of revascularization, and risk of reperfusion injury and hemorrhagic transformation. More large-scale studies are necessary to clarify blood pressure goals in AIS undergoing EVT, including the role of BPV and cerebral autoregulation. 

## 5. Conclusions

Stroke is a major cause of death and disability worldwide. Although there have been significant advancements in stroke risk factor optimization, acute stroke treatment, and secondary stroke prevention, post-stroke care for improving functional status remains poor. Recovery from EVT after AIS is challenging not only due to the damage of the stroke itself but also the side effects of EVT treatment. Disability is largely treated with time and rehabilitation. Several studies have demonstrated the use of adjunctive therapy in improving outcomes and mitigating the persistent symptoms and impairments seen after stroke treatment. With more insight into the cytotoxic and inflammatory pathophysiology of stroke and ischemia-reperfusion injury, a new era of neuroprotective agents may be underway. Although blood pressure regulation takes the lead in post-stroke optimization, preliminary neuroprotection studies on various cellular agents, free radical agents, neurotransmitter agents, oxygen therapies, and hypothermia protocols may allow for a multi-pronged approach to healing ischemic injury from various mechanisms. In particular, the upcoming ESCAPE-NEXT trial involving nerinetide is one to anticipate, as the design plans to exclude thrombolytics, which were thought to have a drug-drug interaction. Other promising therapies include the pharmacologic agents uric acid and APC, as well as regional hypothermia. Future research on combination therapy with thrombectomy and neuroprotective agents is needed to strengthen efforts to improve life with post-stroke disability.

## Figures and Tables

**Table 1:** Summary of endovascular trials. * Trials were stopped early due to efficacy. Abbreviations: IVT, intravenous thrombolytic; EVT, endovascular thrombectomy; DSA, digital subtraction angiography; BMM, best medical management; ICA, internal carotid artery; MCA, middle cerebral artery; ACA, anterior cerebral artery; mRS, modified Rankin Scale; CT, computed tomography; CTA, computed tomographic angiography; CTP, computed tomography perfusion; MRI, magnetic resonance imaging; MRA, magnetic resonance angiography; MRP, magnetic resonance perfusion weighted imaging; CBF, cerebral blood flow.

Trial	N	Inclusion Criteria	Inclusion LVO	Imaging Modality	Therapeutic Window	Control vs. Intervention	Primary Outcome	Findings
MR CLEAN (2010–2014)	500	NIHSS ≥ 2	Distal ICA, proximal MCA (M1 and M2) or ACA	CT and CTA, MRA or DSA	6 h	IV tPA alone vs. IVT + EVT	90-day mRS	EVT was effective and safe. EVT had higher rates of mRS 0–2 as compared to IV tPA alone (32.6% vs. 19.1%).
ESCAPE (2013–2014)	315	NIHSS > 5	ICA or MCA (M1 and M2)	CT and CTA	12 h	IV tPA alone vs. IV tPA + EVT	90-day mRS	EVT group had improved functional outcomes (59% vs. 29%) and reduced mortality (10.4% vs. 19%) as compared to IV tPA alone.
SWIFT PRIME (2012–2014)	196 *	NIHSS ≥ 8 and <30 TICI 0–1	Intracranial ICA, MCA (M1 only)	CTA or MRA	6 h	IV tPA alone vs. IV tPA + EVT	90-day mRS	Stent retriever EVT with Solitaire had improved functional independence (60% vs. 35%) as compared to IV tPA alone.
EXTEND IA (2012–2014)	70 *	NIHSS ≥ 6 Mismatch > 10 mL and core volume < 70 mL	ICA, MCA (M1 and M2)	CTA and CTP	6 h	IV tPA alone vs. IV tPA + EVT	Reperfusion at 24 h and 3-day reduction in NIHSS	Stent retriever EVT with Solitaire had improved reperfusion, early neurologic recovery, and better functional outcomes.
REVASCAT (2012–2014)	206 *	NIHSS ≥ 8 ASPECTS CT > 7 or MR > 6	ICA, MCA (M1 only)	CT or MRI	8 h	BMM alone vs. BMM + EVT	90-day mRS	Stent retriever EVT with Solitaire has been shown to have better functional independence (43.7% vs. 28.2%).
DAWN (2014–2017)	206	NIHSS ≥ 10 Age and NIHSS based core volume	ICA, MCA (M1 only)	CT/CTA/CTP or MRI/MRA	6–24 h	BMM alone vs. BMM + EVT	90-day mRS	EVT improved functional outcomes in an extended therapeutic window.
DEFUSE 3 (2016–2017)	182	NIHSS > 6 Core volume < 70 mL, penumbra volume ≥ 15	Proximal ICA or MCA	CT/CTA/CTP or MRI/MRA/MRP	6–16 h	BMM alone vs. BMM + EVT	90-day mRS	EVT improved functional outcomes in an extended therapeutic window.
ATTENTION (2021–2022)	340	NIHSS ≥ 10	Basilar artery	CT and CTA, MRA or DSA	12 h	BMM alone vs. BMM + EVT	90-day mRS	EVT for basilar artery LVO was superior to BMM with improved functional independence (46% vs. 23%) and reduced mortality (37% vs. 55%).
BAOCHE (2015–2019)	217 *	NIHSS ≥ 10	Basilar artery	CT and CTA or MRI	6–24 h	BMM alone vs. BMM + EVT	Symptomatic ICH at 24 h and 90-day mortality	EVT for basilar artery LVO had better rates of functional independence at risk of more ICH and complications.
RESCUE Japan LIMIT (2018–2021)	203	NIHSS ≥ 6 ASPECTS 3–5	ICA, MCA (M1 only)	CT and CTA or MRA	6–24 h	BMM alone vs. BMM + EVT	90-day mRS	EVT had better functional independence compared to BMM but was at risk of higher rates of ICH.
ANGEL ASPECT (2020–2023)	456	NIHSS 6–30 ASPECT 3–5 Core volume 70–100 mL	Intracranial ICA, MCA	CT and CTA or MRA	24 h	BMM alone vs. BMM + EVT	90-day mRS	EVT had better functional independence compared to BMM but was at risk of higher rates of ICH.
SELECT2 (2019–2023)	352 *	ASPECTS 3–5 CTP CBF < 30% or MRI core volume ≥ 50 cc	Distal ICA, MCA (M1 only)	CT and CTP or MRI	24 h	BMM alone vs. BMM + EVT	90-day mRS	EVT had better functional independence compared to BMM (20% vs. 7%), but at was risk of higher rates of ICH.

**Table 2:** Overview of neuroprotective therapies.

Trial	Neuroprotective Therapies	Mode of Action	Findings
URICO-ICTUS: Phase II/III (2011–2013)	Uric Acid	Antioxidant Downregulation of VEGF-A	Significant improvement in functional outcomes (mRS 0–2) at 90 days compared to placebo in patients receiving IVT followed by EVT.
RHAPSODY: Phase II (2015–2017)	3K3A-APC	Activation of PAR-1, leading to neurogenesis, anti-apoptosis, anti-inflammatory effects	Dose-limiting toxicity for the highest dose (540 μg/kg) was not statistically different from placebo. Reduction in intracerebral hemorrhage (ICH) rates compared to placebo.
ESCAPE-NA1: Phase III (2017–2019)	Nerinetide	Downregulation of excitotoxic cascade via PSD-95 inhibition	Significant improvement in functional outcomes (mRS 0–2) at 90 days compared to placebo in the treatment group that did not receive tPA. 7.5% absolute risk reduction in mortality.
SAFE-TPA: Phase II (2016–2017)	Otaplimastat	Downregulation of MMP Antioxidant	No significant difference in the incidence of parenchymal hematoma compared to the placebo.
I-STROKE: Phase II (2011–2014)	Imatinib	Downregulation of PDGF-alpha	Significant improvements in the mean NIHSS scores with high-dose imatinib compared to controls before and after adjustment for thrombectomy.
SAVER-I: Phase I (2013–2015)	Verapamil	Reduction in calcium-mediated apoptosis	No patients met the primary safety endpoint (i.e., significant ICH).
BAST: Phase III (2018–2022)	Butylphthalide (NBP)	Improvement in microcirculation BBB protection Reduction in mitochondrial dysfunction	Significant improvement in functional outcomes (mRS 0–2) at 90 days compared to placebo.
TASTE: Phase III (2015–2016)	Edaravone dexborneol	Antioxidant	Significant improvement in functional outcomes (mRS 0–1) at 90 days compared to edaravone alone.
SONIC: Phase II (2016–2020)	Nelonemdaz (Neu2000)	NMDA receptor subtype 2B selective antagonist Antioxidant	Favorable trend but no statistically significant difference in the proportion of patients achieving mRS 0–2 at 12 weeks.
APRIL: Phase Ib/IIa (2020–2022)	ApTOLL	TLR4 antagonist	Not sufficiently powered to determine efficacy, but a 0.2 mg/kg dose reduced mortality at 90 days.
Safety, feasibility, and potential efficacy of intraarterial selective cooling infusion for stroke patients treated with mechanical thrombectomy (2015–2017)	Regional Hypothermia	Reduction of basal metabolic rate	No adverse events were associated with intraarterial cold saline infusion in combination with EVT. Significant reduction in final infarct volume but no difference in functional outcomes.
Protective roles of intra-arterial mild hypothermia and arterial thrombolysis in acute cerebral infarction (2016)			Significant reduction in mean infarct volumes and neurological deficits compared to normothermia.
RESIST Trial (2018–2022)	Remote Ischemic Conditioning (RIC)	Strengthening endogenous mechanisms to build tolerance against more severe ischemia	No significant difference in mRS scores at 90 days.
RICAMIS Randomized Clinical Trial (2018–2021)			Significant improvement in functional outcomes (mRS 0–1) at 90 days compared to usual care.
Adjuvant High-Flow Normobaric Oxygen after Mechanical Thrombectomy for Anterior Circulation Stroke: A Randomized Clinical Trial (2017–2019)	Normobaric Oxygen (NBO)	Increase in oxygen level of ischemic penumbra	Significant reduction in 90-day mortality and infarct volume compared to controls in patients receiving NBO therapy for 6 h after recanalization.

## Data Availability

No new data were created or analyzed in this study. Data sharing is not applicable to this article.
